# Prevalence of Blood-Borne Viruses in Health Care Workers of a Northern District in Pakistan: Risk Factors and Preventive Behaviors

**DOI:** 10.1155/2016/2393942

**Published:** 2016-07-25

**Authors:** Muhammad Zuhaib Khan, Shahab Saqib, Sayed Irtiza Hussain Shah Gardyzi, Javaria Qazi

**Affiliations:** ^1^Department of Biotechnology, Quaid-i-Azam University, Islamabad 44000, Pakistan; ^2^National Institute for Biotechnology and Genetic Engineering, Faisalabad 38000, Pakistan; ^3^The Biotech Labs and Research Centre, Islamabad 44000, Pakistan

## Abstract

*Background*. Blood-borne viral infections like viral hepatitis are highly prevalent in Pakistan. There is also a potential threat of human immunodeficiency virus (HIV) spread in the country. Health care workers (HCWs) are a high risk population for acquiring such viral infections and potential spread to the patients. This study aimed to determine the frequency of three blood-borne viruses: HCV, HBV, and HIV in HCWs of district Malakand in northern Khyber Pakhtunkhwa (KPK) province of Pakistan. Moreover, risk factors and preventive behaviors among HCWs were investigated in detail.* Materials and Methods.* Prevalence was investigated using serological assays followed by real time polymerase chain reaction (RT-PCR) based characterization. A total of 626 health care workers working at 17 different health care units, belonging to 6 different job categories, were included in this study.* Results.* HIV was not detected in the HCWs while rate of prevalence of HCV and HBV was far less (0.8 % and 0.64 %, resp.) as compared to general population (4.7%–38%). The majority of HCWs were aware of the mode of spread of these viruses and associated risk factors. Needle stick injury was found to be the most important risk factor for possible acquisition of these infections.

## 1. Introduction

The prevalence of transmissible blood-borne viral infections is quite high in the developing countries [1]. In Pakistan both hepatitis C virus (HCV) and hepatitis B virus (HBV) infections are highly prevalent and there is a considerable threat of human immunodeficiency virus (HIV) spread across the country. HCWs who are constantly in contact with human blood products, infected individuals, and laboratory equipment are at high risk of acquiring these infections [2]. In case of hepatic viruses HCV and HBV most of the cases remain unnoticed because of their asymptomatic nature in the initial phase of infection [3]. Both these viruses have contributed in causing major dreadful liver infections around the globe. According to an estimate there are 200 million chronic carriers of HCV and 350 to 400 million chronic carriers of HBV [4].

Prevalence is the key that provides the information about the spread of a disease in a certain area, the possible risk factors through which infection can be acquired, and treatment response to the infection [5, 6]. In Pakistan, the prevalence of HCV infection in the general population is quite high (4.7%–38%) as compared to HBV prevalence (2.5%). A major contributor to the low prevalence of HBV infection is the availability of vaccine [7–10].

Published data is available about the prevalence of these three blood-borne viruses in different areas and population groups of Pakistan. But almost no data is available on the prevalence of these viral infections in health care workers (HCWs). This study was aimed at determining the prevalence of blood-borne viral infections in HCWs. Moreover, based upon a carefully designed questionnaire each HCW was personally interviewed in order to evaluate knowledge about blood-borne diseases, risk factors, and preventive behaviors. The present study selected a population of HCWs working at health care facilities of district Malakand of KP province in Pakistan. This study gives a very compact and useful knowledge about the rate of infections in HCWs of this area, elaborates preventive behaviors, and highlights important risk factors for acquisition of these viruses.

## 2. Materials and Methods

### 2.1. Plan of Work and Study Design

Our work was comprised of a cross-sectional study of HCWs population in a northern district (Malakand), of Khyber Pakhtunkhwa (KP) province in Pakistan. The study included three types of health care centers: hospitals, basic health units, and rehabilitation centers. There are five public hospitals in district Malakand including one district head quarter hospital (DHQ) and four civil hospitals which were all included in this study. Other than hospitals, six basic health units and six rehabilitation centers were also included. HCWs (most of them exposed to blood or blood products) in these three types of health care centers were approached and requested to participate in this investigation. All available 626 HCWs participated in the study. A comprehensive questionnaire based interview was conducted with all participants. The questionnaire included personal information, previous history, knowledge, attitudes, and work practices related to occupationally transmitted blood-borne viral infections. Data was also recorded in domains of safe injection practices, standard precautions to prevent occupational infection, and preventive measures including HBV vaccination. This study was executed from March 2014 to November 2014. It was a collaborative project of the Molecular Virology Lab, Biotechnology Department, Quaid-i-Azam University, Islamabad, and The Biotech Labs and Research Centre, Blue Area, Islamabad, Pakistan. Institutional ethics review board approval was obtained for this study and sampling was done with informed consent.

### 2.2. Collection of Blood and Serological Testing

Blood samples were taken from all HCWs after getting verbal informed consent. Venous blood (5 cc) samples were obtained from each participant using sterilized syringes and stored at room temperature in gel tubes. Samples were centrifuged at 4000 rpm for 15 min by using Eppendorf centrifuge to separate serum. A total of 626 samples were initially screened for anti-HIV, anti-HCV, and HBs-Ag by immunochromatographic technique (ICT) procedures using strips obtained from Standard Diagnostics (SD), Germany. All 626 samples were also tested through ELISA for the presence of anti-HCV and HBs-Ag (BIOKIT, S.A., Barcelona, Spain) according to the manufacturer's instructions but not for anti-HIV.

### 2.3. Universal and Genotype Specific PCR Procedures

All the ELISA positive samples for HBV and HCV were subjected to universal and genotype specific PCR after total nucleic acid extraction using Vivantis kit (Malaysia, cat # GF-RD 300). As described by Naito and coworkers a two-step method was adopted for HBV universal PCR and genotyping [11]. For HCV samples 5′ UTR was reverse-transcribed and amplified with the help of gene specific primers as described earlier [12]. Real time PCR thermal cycler (MiniOpticon, BioRad) and SYBR Green Master Mix were used for amplifications (QuantiFast, USA, cat # 204154).

### 2.4. Biochemical Tests and Liver Ultrasound

The liver function tests (LFTs) were performed for nine PCR positive (5 HCV and 4 HBV) HCWs including alanine aminotransferase (ALT), alkaline phosphatase (ALP), and bilirubin using kits and absorbance was measured through Shimadzu UV-Visible Double Beam Spectrophotometer 1700 Pharma (Japan) as described in the manufacturer's manual. Liver ultrasound for these HCWs was performed commercially.

### 2.5. Statistical Analysis

Data were coded, validated, and analyzed using the Statistical Package for the Social Sciences (SPSS), version 18.0 (SPSS Inc., Chicago, IL, USA). The frequency, percentage, arithmetic mean, and mode are used to present the data.

## 3. Results

### 3.1. Description of the Sampling Population

The sampling population was comprised of all available (626) HCWs working at different health care units in different departments throughout district Malakand. Each HCW was interviewed personally by the first author. The majority of HCWs in the sample population were males and due to this reason HCWs included in the study also were mostly males (*n* = 491, 78.43%) as compared to female HCWs (*n* = 135, 21.57%). The majority of health care workers were residents of district Malakand (*n* = 618, 98.72%), and the rest were from other areas (*n* = 08, 1.3%). HCWs included in this study belonged to six different job categories including doctors (*n* = 71, 11.34%), nurses (*n* = 72, 11.50%), ward boys/girls (*n* = 184, 29.39%), technicians (*n* = 163, 26.03%), midwives (*n* = 51, 8.14%), and sanitary workers (*n* = 85, 13.75%) as depicted in Table 1. The age of the subjects ranged from 20 to 60 years with an average of 40.18 years. Subjects were divided into four age groups: 21–30 (*n* = 118, 18.85%), 31–40 (*n* = 254, 40.58%), 41–50 (*n* = 187, 29.87%), and 51–60 (*n* = 67, 10.70%).

### 3.2. Knowledge, Work Practices, and Preventive Behaviors

**Figure 1:**
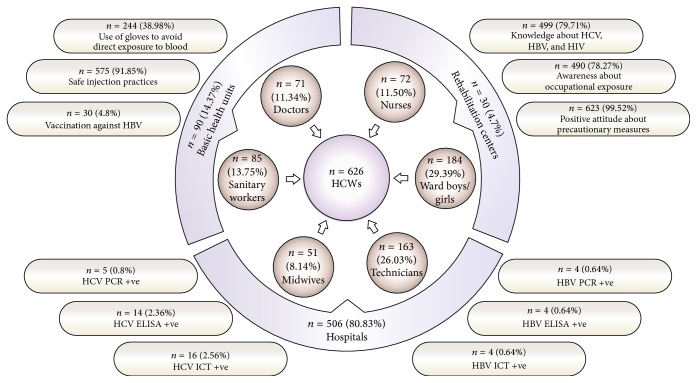
Description of study population along with preventive behaviors and rates of infection.

The majority (79.71%, *n* = 499/626) of HCWs were aware of major blood-borne diseases (HIV, HBV, and HCV) as shown in Figure 1. Out of these a large percentage of HCWs (*n* = 490/626, 78.27%) had a good understanding of the procedures and work practices involving high risk of occupational exposure. The majority of HCWs were aware of all safe injection practices that may protect them (*n* = 575/626, 91.85%) and all standard isolation precautions to prevent occupational transmission. The majority (*n* = 623/626, 99.52%) of HCWs had a positive attitude towards routine testing for blood-borne pathogens as a preventive measure. However in spite of awareness about the risk of occupational exposure only a small fraction of HCWs (*n* = 30/626, 4.8%) had received vaccination against HBV in the previous 5 years. Unfortunately use of gloves in order to avoid direct contact with blood or blood products was not a common practice. The majority of the HCWs were reluctant to use gloves while performing routine activities. A total of 244 subjects out of 626 (38.98%) were using gloves in routine.

### 3.3. Blood-Borne Viral Infections Confirmed by Real Time PCR

When screened through ICT none of the subjects was found positive for anti-HIV. No other diagnostic test was applied to samples for HIV after initial screening with ICT. A total of 9 HCWs were PCR confirmed for the presence of viral hepatitis either HCV or HBV. Five HCWs were PCR positive for HCV and four for HBV. As a result of this study we report a low rate of prevalence of viral hepatitis in HCWs of district Malakand: 0.8% in case of HCV and 0.64% for HBV. None of the HCWs was positive for the presence of HIV (tested through ICT only). We did not find any coinfections in this study, that is, presence of multiple viruses in a single individual.

### 3.4. Seroprevalence of Anti-HCV Antibodies in HCWs

To check for the presence of anti-HIV only ICT procedures were employed during this study. However in case of HCV and HBV three types of diagnostic tests including ICT, ELISA, and PCR were performed. All samples were initially screened by ICT and then all samples whether positive or negative for hepatitis were checked by ELISA for further confirmation. All samples which were negative for HCV or HBV using ICT devices were also negative when checked by ELISA, showing no false negative results by ICT.

The confirmation of positive results of ICT when tested by ELISA and RT-PCR presented a different situation for HCV and HBV. In case of HBV four subjects were seropositive for HBs-Ag when tested through ICT and also by ELISA. Results remained the same when RT-PCR technique was used to amplify viral DNA from seropositive samples. However in case of HCV 16 subjects were anti-HCV positive when tested by ICT and the number reduced to 14 when the same samples were tested by ELISA. Further confirmation of these 14 samples by RT-PCR using universal primers for HCV detection revealed the presence of viral RNA in only five subjects.

### 3.5. Genotyping of HCV and HBV

Genotyping of HCV positive samples revealed the presence of 3a as the dominant genotype in case of HCV where 4 out of 5 samples belonged to this group, while the remaining sample was of the 3b genotype. In case of HBV, all 4 samples belonged to the D genotype.

### 3.6. Blood Biochemistry and Liver Ultrasound

Infected HCWs had elevated levels of alanine aminotransferase, whereas alkaline phosphatase and serum bilirubin were in normal range. Liver ultrasound showed normal liver size with no signs of cirrhosis and fibrosis. Biochemical tests and liver ultrasound validated the possibility of acquiring these infections in subjects after joining their jobs as HCWs.  Table 2 provides details of these results.

### 3.7. Risk Factors for Acquiring Infections at Work

Exposure to infected blood products and needle stick injuries were defined as the major risk factors for acquiring viral hepatitis as a result of our study. Case history and detailed investigation suggests that none of the subjects had a previous history of viral hepatitis before joining their job as an HCW. Moreover all infected HCWs had history of needle stick injuries and exposure to blood while performing their job.

## 4. Discussion 

Our main finding of the study is that in spite of being a high-risk group HCWs of district Malakand showed a low prevalence (0.8% in case of HCV and 0.64% for HBV) of hepatic viruses as compared to the general population of the country [9, 13, 14]. No case of HIV was reported among these HCWs. Epidemiological studies on HCV in different cities of Pakistan report a high rate of prevalence ranging from 1.8% in Karachi to 23.8% in Gujranwala [8, 13, 15]. District Malakand is located in the north of the country in the KP province. Epidemiological data from KP province suggest high prevalence rates of HCV [16, 17]. Published data on epidemiology of hepatic viruses in the general population of district Malakand is not available; however according to our unpublished data, the occurrence of HCV infection in the general population of district Malakand is quite low 1.3% as compared to other districts in province KP. HCWs are at high risk to acquire these viral infections after they come in contact with infected patient or blood products. Due to low prevalence in the general population the chance of acquiring infection by HCWs during contact is minimized. A study on prevalence of HBV in internally displaced population mainly belonging to Malakand reveals alarmingly high rates of seroprevalence of 21% based upon ELISA [18]. When prevalence of hepatitis in HCWs of Malakand district is compared to the general population in Malakand or KP province and other parts of the country it is concluded that HCWs have a very low prevalence rate.

With a significant number of samples this study provides substantial data about the prevalence of blood-borne pathogens in HCWs of a selected area of Pakistan. Moreover, the sampling population was comprised of all available HCWs, reducing possibility of selection bias. On the other hand several factors oppose such selection bias. The very first evidence against selection bias is that not few but all the available HCWs working in different health units having different level of precautionary measurements participated in the study. Secondly the proportion of participants who indicated past percutaneous exposure and the relatively large number of recent accidental exposures (as shown in Figure 1) point towards participation of a high-risk population.

The theoretical risk of being infected with a blood-borne pathogen can be calculated from the prevalence of infection in the general population in that area, the frequency of exposure to infected products, and the risk of transmission at each exposure. These factors mentioned in Figure 1 provide an indication of the percentage of occurrence of viral hepatitis in health care workers and elaborate major risk factors for acquiring these infections in hospital environment. We did find a positive association between blood exposures, specially needle stick injury, and HCV/HBV infection.

Three types of diagnostic tests including ICT, ELISA, and PCR were performed for hepatitis B, hepatitis C, and HIV detection in the HCWs in the present study. Results indicate that ICT may give false positive results due to cross reaction with other antibodies or antigens that are also present in the test specimen. However in the experiments conducted during this research work no false negative results were recorded. It is interesting to note that in case of HCV not all the samples which were seropositive with ELISA were PCR positive. ELISA in case of HCV was performed against HCV antibodies in the serum and not the virus itself. That is why the ELISA and PCR results differ from one another: high seropositivity as compared to low PCR confirmation. In case of HBV, ELISA confirmed the HBs-Ag, so no difference was recorded in ELISA and PCR results. Seropositivity is not always indicative of the presence of infection in the body but may result due to prior exposure to the virus which may or may not persist in the body and cause infection or sometimes the virus is present but the viral load in the body is not sufficient to elicit the infection. That is why the antibody response is observed in many cases. Detection of the antibodies reflects the host response but could not reflect viral replication and infection confirmation [19, 20].

Level of awareness about blood-borne pathogens and risk of occupational exposure was high among HCWs of district Malakand. The majority were in favor of routine testing and were using safe injection practices. However use of glove was not common in procedures other than injection. Also vaccination against HBV was not adopted as a preventive measure in the majority of HCWs. Percutaneous blood exposure was identified as the principal risk factor for acquiring these infections at work. It is suggested that more facilities and proper training are required to improve and implement safe practices and encourage vaccination against HBV in HCWs.

## Figures and Tables

**Table 1:** Gender-, occupation-, and agewise distribution of the sample population.

Occupation					Male/female	Total
Technicians	27/11	78/8	35/1	3/0	143/20	163
Ward boys	37/0	61/2	64/1	18/1	180/4	184
Midwives	0/1	0/8	0/20	0/22	0/51	51
Nurses	2/13	13/27	8/9	0	23/49	72
Sweepers	20/0	21/1	25/2	15/1	81/4	85
Doctors	6/1	30/5	23/1	5/0	64/7	71
*Age*	*20–30*	*31–40*	*41–50*	*51–60*	*—*	*626*

**Table 2:** Blood biochemistry and liver ultrasound for different infection patterns.

Type of infection	Genotype	Number of subjects	Alanine aminotransferase mean	Alkaline phosphatase mean	Serum bilirubin mean	Liver ultrasound
HBV	D	4	36.25 ± 3.30	199 ± 5.89	0.68 ± 0.17	Normal

HCV	3a	4	49.25 ± 15.22	205.25 ± 16.58	1.07 ± 0.61	Normal
	3b	1	39	196	0.6	Normal
